# Novel Nanotechnology Approaches to Overcome Drug Resistance in the Treatment of Hepatocellular Carcinoma: Glypican 3 as a Useful Target for Innovative Therapies

**DOI:** 10.3390/ijms231710038

**Published:** 2022-09-02

**Authors:** Monica Mossenta, Davide Busato, Michele Dal Bo, Paolo Macor, Giuseppe Toffoli

**Affiliations:** 1 Experimental and Clinical Pharmacology Unit, Centro di Riferimento Oncologico di Aviano (CRO), IRCCS, 33081 Aviano, Italy; 2 Department of Life Sciences, University of Trieste, 34127 Trieste, Italy

**Keywords:** hepatocellular carcinoma, glypican 3, nanomedicine, drug delivery, polymeric nanoparticles, targeted therapy

## Abstract

Hepatocellular carcinoma (HCC) is the second most lethal tumor, with a 5-year survival rate of 18%. Early stage HCC is potentially treatable by therapies with curative intent, whereas chemoembolization/radioembolization and systemic therapies are the only therapeutic options for intermediate or advanced HCC. Drug resistance is a critical obstacle in the treatment of HCC that could be overcome by the use of targeted nanoparticle-based therapies directed towards specific tumor-associated antigens (TAAs) to improve drug delivery. Glypican 3 (GPC3) is a member of the glypican family, heparan sulfate proteoglycans bound to the cell surface via a glycosylphosphatidylinositol anchor. The high levels of GPC3 detected in HCC and the absence or very low levels in normal and non-malignant liver make GPC3 a promising TAA candidate for targeted nanoparticle-based therapies. The use of nanoparticles conjugated with anti-GPC3 agents may improve drug delivery, leading to a reduction in severe side effects caused by chemotherapy and increased drug release at the tumor site. In this review, we describe the main clinical features of HCC and the common treatment approaches. We propose the proteoglycan GPC3 as a useful TAA for targeted therapies. Finally, we describe nanotechnology approaches for anti-GPC3 drug delivery systems based on NPs for HCC treatment.

## 1. Introduction

**Figure 1:**
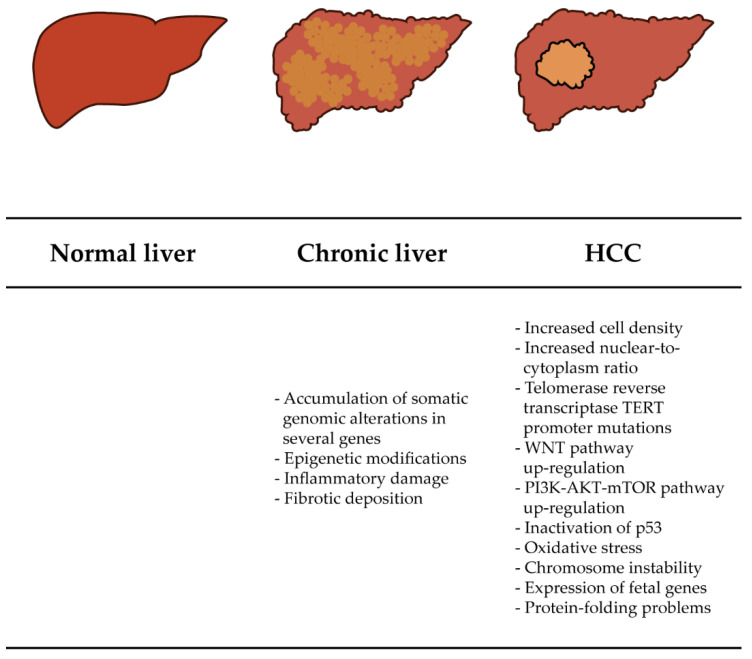
HCC pathogenesis. Chronic liver diseases involve changes leading from a normal and healthy liver to a state of cirrhosis presenting inflammatory damage and fibrotic tissue. The continuous inflammation caused by cirrhosis produces somatic genomic alterations and epigenetic mutations, which progress to HCC. HCC cells present an increased nuclear-to-cytoplasm ratio. Furthermore, HCC is characterized by several alterations in the proliferation pathways, protein expression and folding, and oxidative stress.

Hepatocellular carcinoma (HCC) is the second most common fatal tumor with a 5-year survival rate of 18% and accounts for nearly 90% of all liver tumors [1,2]. The development of HCC depends on several factors and proceeds in a multistep process [1,2]. Most HCC cases develop against a background of chronic liver disease, in which ongoing liver injury and anomalous regeneration lead to a state of inflammation and progressive liver fibrosis that predispose patients to consequent cirrhosis and neoplasia [1,2]. Major risk factors for the development of HCC include chronic infection with hepatitis B virus (HBV) or hepatitis C virus (HCV), heavy alcohol consumption, liver cirrhosis, exposure to environmental or dietary carcinogens (e.g., aflatoxin B1), genetic and metabolic liver diseases (e.g., nonalcoholic steatohepatitis, hereditary hemochromatosis), and other liver injury [2,3,4]. Molecular alterations occur at different stages of HCC development and in most cases are triggered by a specific etiologic factor [5,6,7,8]. Some of these alterations involve the re-expression of fetal genes, mutations in telomere reverse transcriptase (TERT) promoter, TP53 mutations, problems in protein folding, chromosome instability, oxidative stress, and activation of three signaling pathways related to cell proliferation: WNT-β-catenin, RAS-Raf-MAPK, and PI3K-AKT-mTOR (Figure 1) [2,9].

Decision making for the treatment of HCC is primarily based on the Barcelona Clinic Liver Cancer (BCLC) clinical classification. Early stage cancers are treated with curative intent surgical resection, liver transplantation, or local ablation (e.g., radiofrequency, microwave). Locally advanced HCC is typically treated with chemoembolization, radioembolization, or stereotactic ablative radiotherapy, which are often used either as a bridge to transplantation or to defer systemic therapy [1,2,10]. Unfortunately, the overall prognosis for patients with HCC is dramatically poor. Most HCC patients are diagnosed at late stages when curative treatments are not available, making systemic therapy the only clinical option. Even when surveillance programs contribute to early diagnosis and curative treatments, the majority of HCC patients are ultimately treated systemically [2,11]. Standard chemotherapic regimens are poorly effective in HCC and minimally prolong patient survival. This is due to the complexity of the tumor type, with its genomic and molecular heterogeneity that changes and mutates over time when exposed to selective pressures, leading to anticancer drug resistance [1,2,12,13,14,15,16,17,18,19,20]. Therefore, current cancer research in HCC, as in other malignancies, is focused on the development of innovative strategies and antitumor agents to overcome anticancer drug resistance and achieve better efficacy [21,22,23,24,25,26,27,28,29,30]. Targeted strategies are a potentially effective option for the introduction of innovative therapies [31,32,33]. In this context, ligand-conjugated nanoparticle-based targeted therapies can be proposed, with ligands targeting specific tumor-associated antigens (TAAs) [32,34,35]. Glypican 3 (GPC3) is a member of the glypican family, heparan sulfate proteoglycans bound to the cell surface via a glycosylphosphatidylinositol (GPI) anchor [36,37]. GPC3 is not expressed in most adult tissues, whereas its expression is upregulated in HCC at both the transcript and protein levels compared to normal liver and premalignant lesions [38,39,40,41,42,43,44,45,46,47,48,49]. The high levels of GPC3 detected in HCC and the absence or very low levels in normal and non-malignant liver make GPC3 a promising TAA candidate for ligand-conjugated nanoparticle-based targeted therapies.

In 1959, Nobel Prize-winning physicist Richard P. Feynman gave the famous lecture “There’s Plenty of Room at the Bottom” at the California Institute of Technology. He argued theoretically about the possibility of making things at the atomic level and how these manufactured tiny objects respond to different physical laws than the large-scale copies, giving them different properties [50]. Based on this inspiring speech, the field of nanotechnology emerged and grew over time.

Dr. Feynman’s foresight has been confirmed, as nanomaterials have new properties that are different from those of large-scale materials. Some examples include insoluble substances that become soluble or chemically inert metals that become catalysts at the nanoscale [51]. The ability to manipulate these and other physicochemical and biological properties has given nanomaterials an important place in technological development [52]. Nanomaterials can be classified into four different categories depending on the material used: carbon-based, inorganic-based (characterized by metals and metal oxides), composite-based (multiphase nanoparticles and nanostructured materials), and organic-based (i.e., micelles, dendrimers, liposomes, and polymeric nanoparticles) [52].

In the field of oncology, several therapies have been developed based on nanoparticles whose properties have been tuned with the aim of improving drug delivery to the tumor site, with a consequent overcoming of the impairments caused by chemotherapy and radiotherapy [53,54]. The usually low drug dose used for therapy is a significant aspect that determines acquired resistance; therefore, increased drug delivery could improve the antitumor effect, preventing the establishment of drug resistance [55]. It has been shown that nanoparticles loaded with doxorubicin and coated with anti-transferrin receptor were able to overcome doxorubicin-resistant MDA-MB-231 cells, increasing NPs’ internalization and, consequently, cellular drug uptake [56]. Gan and colleagues increased sorafenib delivery using polymeric NPs coated with an anti-GPC3 antibody promoting cellular apoptosis and in vivo tumor growth inhibition [57]. Polymeric NPs loaded with tamoxifen have been used to exploit the overexpression of HER-2 in multi-drug resistance MCF-7, inducing cell apoptosis through an increase in cancer-cell-targeted delivery and sustained drug released [58]. Along with their advantages, nanoparticle-based devices for cancer diagnosis and treatment also have some limitations [33,59,60,61]. Therefore, despite the remarkable advances in the field of nanomedicine, there are still problems to overcome, such as the difficulty in categorizing and evaluating the safety, cost-benefit considerations, and the lack of regulatory guidelines specific for these nanomaterials. Consequently the standard oncological treatments still rely on conventional methods such as surgery, radiotherapy, and chemotherapy [60,61].

In this review, we describe the main clinical features of HCC and the common treatment approaches. We propose the proteoglycan GPC3 as a useful TAA for targeted therapies. Finally, we provide a description of recently proposed nanotechnology approaches for drug delivery systems based on nanoparticles for active targeted therapies for HCC treatment.

### 1.1. Diagnosis of HCC

In patients enrolled in surveillance programs, HCC is diagnosed at early stages when the disease is not yet symptomatic. In contrast, in patients outside these programs, the diagnosis is usually made at late stages when the symptoms arise [2]. Diagnosis is made by imaging techniques (e.g., multiphase computed tomography, magnetic resonance imaging, contrast-enhanced ultrasound) based on the particular vascular pattern during hepatocarcinogenesis or by biopsy [2,62,63]. Imaging techniques have a high degree of reliability when used in cirrhotic patients, in whom the results may highlight HCC features. Biopsy, on the other hand, is recommended in the early stages of HCC lesions when the radiological result may be controversial [2,62,63]. Although the sensitivity of biopsy ranges from 70% to 90%, it is not an ideal gold standard due to sampling variability that correlates with minimal morphologic changes in liver tissue in the early stages of carcinogenesis [2,62,63]. The most used serum biomarker is alpha-fetoprotein (AFP), as persistently high serum levels of this protein are a risk factor for HCC. However, only 10–20% of early HCC have abnormal AFP levels, and HBV or HCV infection may affect the diagnostic accuracy of serum AFP quantification [63]. The combination of ultrasound and AFP increases the detection rate but also the number of false-positive results [62]. AFP and the other serum biomarkers used, such as des-γ-carboxyprothrombin DCP and AFP-L3 fraction, are often associated with advanced disease stages, so a diagnostic serum biomarker is needed in the early stages [2].

### 1.2. HCC Treatment

**Figure 2:**
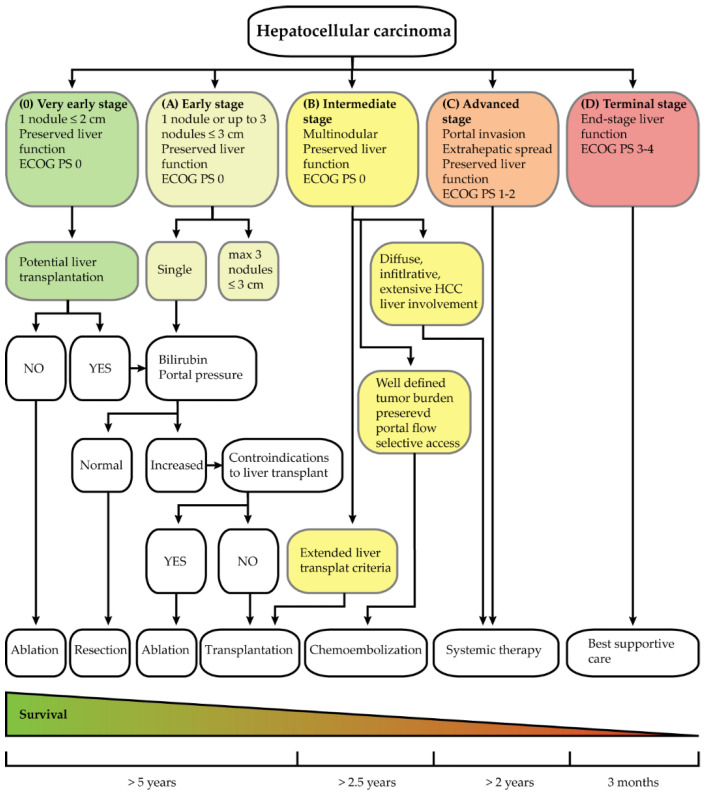
Barcelona Clinic Liver Cancer (BCLC) staging and treatment strategy. The scheme shows the classification of HCC and the possible treatments for each stage. Figure based on the BCLC group guidelines of 2022.

Based on tumor characteristics such as tumor stage and liver function impairment, patients are usually stratified using BCLC staging to assess their prognosis and select the appropriate treatment (Figure 2). The latest update was released in 2022 by the BCLC group in the *Journal of Hepatology* [2,10,62,63]. BCLC staging is based on three types of prognostic variables: tumor status (number and size of nodes, vascular invasion, extrahepatic spread), liver function (Child–Pugh class, albumin, bilirubin, ascites, portal hypertension), and overall tumor-related health status (Eastern Cooperative Oncology Group (ECOG) classification and presence of symptoms) [63].

In early stage HCC (BCLC 0 and A) with solitary tumor, without portal hypertension, and with well-preserved liver function, both surgical resection and local ablation are possible, with survival rates at 5 years of 50–90% [2,63]. Surgical resection is the first choice in the treatment of HCC patients without cirrhosis who are unsuitable for ablation and allows assessment of the early recurrence risk thanks to the possibility of direct histological examination [2,62,63]. Local ablation is used to treat patients unsuitable for surgery and causes tumor necrosis by changing the temperature or injecting chemicals [2,62,63]. Liver transplantation is the best option for patients who cannot undergo surgical resection or ablation and have BCLC A with a single tumor nodule ≤ 5 cm or up to 3 nodules ≤ 3 cm in size without vascular invasion [2,62]. The median survival rate is 70% at 5 years, with a recurrence rate of 10% and a 10-year survival rate of more than 50% [2]. A phase III strong randomized controlled trial (NCT01963429) compared proton beam radiotherapy versus radiofrequency ablation for local efficacy and clinical outcomes in patients presenting recurrent HCC [64]. Proton beam radiotherapy showed better feasibility and a non-inferior local progression-free survival compared to radiofrequency ablation in both per-protocol and intention-to-treat populations, suggesting it may be a promising treatment option for small HCC [64].

Intermediate-stage HCC (BCLC B) has multinodular disease with preserved liver function and without tumor-related symptoms, vascular invasion, and extrahepatic spread [2]. Three subgroups are made to better define patient treatment [10]. In the first one, patients presenting well-defined HCC nodules undergo liver transplantation if they meet the “Extended Liver Transplant criteria”. In the second group, patients are treated with transarterial chemoembolization (TACE), which is the injection of an emulsion of Lipidol and a chemotherapeutic agent, usually doxorubicin, into one or more branches of the hepatic artery supplying the tumor nodules. After injection, the emulsion causes both embolization, reducing oxygen delivery, and cytotoxic effect due to the activity of the chemotherapeutic agent [2,10,63,65]. The median overall survival ranges from 26 to 40 months [2,63]. In the third group, patients presenting diffuse, infiltrative, and extensive HCC are treated with systemic therapies [10].

Advanced-stage HCC (BCLC C) is characterized by symptoms related to the tumor, macrovascular invasion, or spread of the cancer outside the liver [2,63]. It is treated with systemic therapies, which are summarized in Table 1.

For end-stage HCC (BCLC D), there are no treatment options, only pain management and nutritional and psychological support, with a median survival of 3–4 months [2,63].

Unfortunately, chemotherapeutic treatments affect not only proliferating cancer cells but also healthy tissues, leading to various adverse effects [53,86]. Moreover, cancer cells can become resistant to chemotherapeutic agents by downregulating the drug uptake mechanism and increasing the drug efflux rate [30,86,87]. Therefore, the development of drug carriers, such as nanoparticles (NPs), capable of transporting drug amounts without causing drug degradation, off-target adverse effects, and acquired resistance in cancer cells is necessary [86,88].

## 2. Tumor-Associated Antigens Useful for HCC Treatment

In the HCC context, several proteins were identified as TAAs such as: AFP, new york esophageal squamous cell carcinoma-1 (NY-ESO-1), synovial sarcoma X breakpoint 2 (SSX-2), melanoma antigen gene (MAGE), midkine (MDK), hypoxia-inducible factor-1α and -2α (HIF-1α and HIF-2α), epithelial cell adhesion molecule (EpCAM), asialoglycoprotein receptor (ASGPR), transferrin receptor 1 (TfR1), folic acid receptor (FR), scavenger receptor class B type I (SR-B1), mucin-1 (MUC1), carcinoembryonic antigen (CEA), prostate-specific membrane antigen (PSMA), tumor endothelial marker 1 (TEM1), phosphatases of regenerating liver-1 and -3 (PRL-1 and PRL3), cluster of differentiation 147 (CD147), roundabout homolog 1 (ROBO1), programmed death-ligand 1 and 2 (PD-L1 and PD-L2), and GPC3 [38,89,90,91,92,93,94,95,96,97,98,99,100,101,102,103,104,105,106,107,108,109,110,111,112,113,114,115,116,117,118,119,120]. AFP protein is detected during embryonic development, but its level decreased after birth. It is a marker of liver pathologies, including malignant lesions such as HCC, showing an increase in its levels during liver diseases [92]. NY-ESO-1 is part of the cancer-testis antigens family and its mRNA expression was detected in 14.1% of HCC tumor tissues [93]. SSX-2 is another member of the cancer-testis antigens, and its mRNA expression was detected in 14/30 HCC tissues but in none of the 30 non-tumor samples [94]. MAGE gene expression was firstly found in melanomas. Their levels were also evaluated in HCC tissue samples, showing a 74% HCC positivity for at least one of the MAGE-1, -2, -3, and -4 genes and 86% positivity for at least one of MAGE-1, -2, -3, -6, -8, -10, -11, and -12; however, no expression was detected in non-malignant liver tissue [95,96]. The heparin-binding growth factor MDK is a glycosylated protein formed by two domains bound one to each other by disulfide bonds. Its expression was found during development and in limited normal adult tissues. MDK is also produced during the oncogenesis of several solid tumors, including HCC, and has a role in cell proliferation, cell migration, angiogenesis, and metastasis [97,98]. PRL-1 and PRL-3 belong to the dual-specificity superfamily and were found overexpressed in HCC and correlated with an aggressive phenotype, poor differentiation, and prognosis [91]. HIF factors are important sensors for the oxygen concentration inside the tumor tissue and present two groups: the α-subunits and the β-subunits. The heterodimerization of HIF-1αβ is important for angiogenesis, proliferation, invasion, and tumor metabolism [87]. High levels of HIF-1α have been observed in 57.1% of HCC tissues while low levels were detected in peritumoral tissues: 5.6%. On the contrary, HIF-2α levels were low in HCC tissues at 13.5% and high in peritumoral regions at 47.6% [121]. A significant decrease in HIF-1α and HIF-2α levels has been observed in HCC cells after treatment with digitoxin and sorafenib coupled with a decrease in cell viability [114]. In the case of AFP, NY-ESO-1, SSX-2, MAGE, MDK, PRL-1 and -2, and HIF-1α and -2α, their localization inside the cellular compartment makes them not ideal targets for a drug delivery approach that exploits external proteins/receptors to redirect NPs towards tumor cells [35].

EpCAM is a transmembrane glycoprotein that is found in many types of epithelial tissues, several stem and progenitor cells such as liver cancer stem cells, and almost 35% of HCC cases [99]. ASGPR is a transmembrane protein made of two subunits: ASPGR1 and ASPGR2. It is abundant in hepatocytes and expressed in the sinusoidal and basolateral HCC cell membranes, showing an expression polarity and zonality [90]. A study performed on 177 HCC and 114 normal or non-cancerous liver samples showed that 75.2% and 88.2% of HCC and normal liver samples were ASGPR1 positive. In addition, ASGPR1 expression in well-differentiated HCC and in poorly differentiated HCC was similar or lower compared to normal tissues, respectively [100]. TfR1 is a protein exploited in iron regulation and cell growth. It is expressed universally among the different cell types and overexpressed in HCC. An increase in TfR1 positivity was detected from early tumor with well-differentiated histology to advanced HCC with poorly differentiated histology, with a 14.3% positivity in non-cancerous tissues [90,101,102]. FR is a GPI-anchored protein presenting two major isoforms: FR-α and FR-β. The first is overexpressed in several cancers, including HCC, while the second is present in tumor-associated macrophages of different cancers, including liver cancer [90,103]. SR-B1 is part of lipoprotein receptors; it is able to bind cholesterol and it is expressed in many mammalian cells and tissues such as macrophages, intestine, endothelial cells, keratinocytes, smooth muscle cells, placenta, and adipocytes and it is widely present in hepatocytes and HCC [90,104]. MUC1 is a glycoprotein, which acts in the modulation of cell adhesion and metastasis. MUC1 is not expressed in normal liver, but progenitor hepatocytes and cancer cells have been found to be positive. Overexpression of MUC1 has been observed in HCC and associated with HCC recurrence and metastasis [105,106]. CEA is a GPI-anchored glycoprotein overexpressed in several solid tumors, with HCC among them, and it is involved in the spread of metastases [107,108]. PSMA is a type II transmembrane protein found to be overexpressed in prostate cancer but also detected in HCC. Among 103 HCC tissue samples, 48% had ≤50% PSMA expression, and 26% had more than 50% in tumor-associated vasculature, but no signal was detected in 5 normal liver tissues [109]. TEM1 (also known as endosialin) is a transmembrane glycoprotein rich in sialic acid belonging to the C-type lectin receptor family [110,111]. TEM1 has weak expression in healthy adult tissues, but it is overexpressed in progressing tumors at the stromal compartment [112]. In HCC, 31.5% of cells were positive, and TEM1 expression was heterogeneous, with regional variation in the specimen’s stroma and higher signal in the invasion region and fibrous capsule. In particular, TEM1 expression was limited to mesenchymal cells, such as stellate cells and myo-fibroblasts, within the tumor [112]. CD147 is a transmembrane glycoprotein present throughout the human body. In HCC, it promotes de novo lipogenesis and glycolysis. It also plays a role in cell proliferation and metastasis. Its overexpression in HCC tissues is correlated with poor prognosis [115,116,117]. ROBO1 is part of the immunoglobulin superfamily, and it has been found to be expressed in 83 out of 98 HCC tissue samples. Its expression has also been detected in tumoral endothelial cells and correlated with a shorter disease-free survival and HCC angiogenesis [118,119]. PD-L1 and PD-L2 are surface proteins acting on the maintenance of peripheral tolerance and negatively regulating T cell functions. PD-L1 and PD-L2 have been found to be overexpressed in 92.6% and 88.9% of HCC tissues, respectively. However, less than 50% of cells in tumor tissues expressed the two proteins [120]. GPC3 is a GPI-anchored proteoglycan that regulates several growth factors such as Wnt. It is expressed in both the liver and kidneys of fetuses but barely expressed in adult tissues (gastric glands 3 samples out of 7, kidney tubules 9/17, testicular germ cells 2/16). Importantly, it is specifically present in HCC tissue [38,90,113]. Among these several valuable TAAs, GPC3 was counted as a promising target protein for diagnosis, treatment, and drug delivery approaches thanks to its specificity for HCC tissue [122,123,124].

## 3. Glypican 3 as Targeting Protein

The glypican family is composed of six members, from glypican 1 (GPC1) to glypican 6 (GPC6), grouped into two subfamilies, which share 25% amino acid identity: glypicans 1, 2 (GPC2), 4 (GPC4), and 6 and glypicans 3 and 5 (GPC5) [36]. The six glypicans share common features: all are heparan sulphate (HS) proteoglycans bound to the cell membrane surface via a GPI anchor [36,37,125,126].

Filmus and colleagues first identified the homologous gene of GPC3 in the rat, named *OCI-5*, in 1988 [127]. The name glypican 3 was first used in 1996 when mRNA studies revealed its overexpression in mesodermal embryonic tissues that usually overgrow in Sympson–Golabi–Behemel syndrome. The name is derived from the homology of this gene with the other members of the glypican family [128].

**Figure 3:**
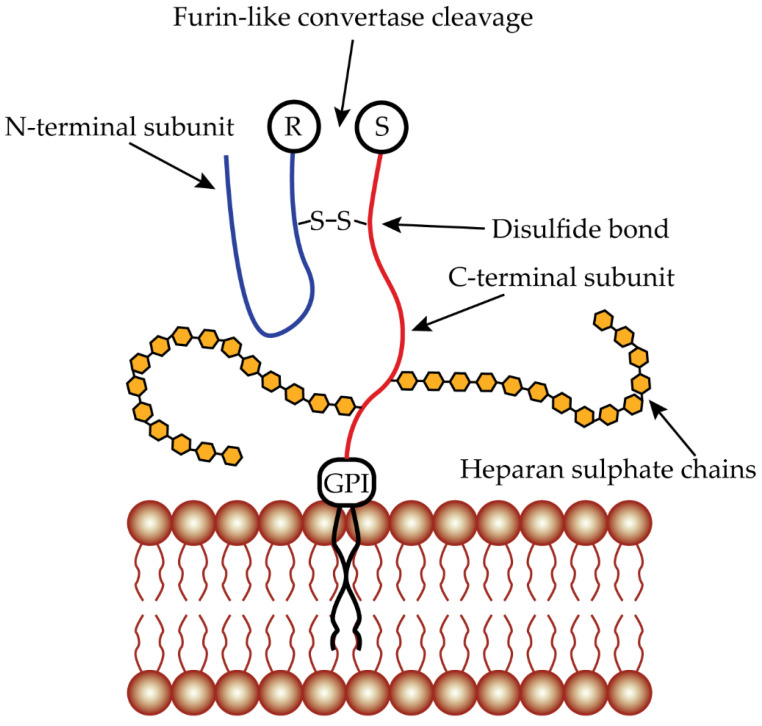
Glypican 3 structure. GPC3 protein presents a structure that similar to the other glypicans. A cleavage site for furin-like convertases is localized between Arg358 and Ser359. Once cleaved, two subunits are formed, bound to each other by a single disulfide bond. In the N-terminal subunit, there is the N-terminal secretory signal peptide while at the C-terminal subunit, there are two sites for the insertion of the HS chains at Ser495 and Ser509 and a GPI anchor at the end.

The human *GPC3* gene is located on the X chromosome (Xq26) and has a length of approximately 500 kb, divided into 8 exons, and encodes the heparan sulfate proteoglycan protein GPC3 [128,129]. It is a 70 kDa protein consisting of 580 amino acids that is cleaved during maturation into 2 subunits that remain linked by a disulfide bond (Figure 3). The cleavage occurs by furin-like convertases between Arg358 and Ser359 to form the N-terminal subunit of 40 kDa and the C-terminal subunit of 30 kDa characterized by 2 HS chains at Ser495 and Ser509 [130,131]. It has three N-glycosylation sites at Asn124, Asn241, and Asn418 [132]. Like the other glypican members, GPC3 also has the COOH-terminal hydrophobic GPI anchor domain at its C-terminus for binding to the cell membrane [36,37,130].

GPC3 is an oncofetal protein involved in signaling pathways correlated with hepatocarcinogenesis and cell proliferation. It has been associated with CD44, a liver cancer stem cell marker, to define the conversion of cirrhotic cells into HCC cancer cells [39,133,134]. GPC3 plays a role in the WNT pathway and likely interacts with Frizzled, leading to accumulation of β-catenin in the cytoplasm and promoting cell proliferation [135,136,137]. GPC3 also interacts with glucose metabolism of HCC and increases the expression of HIF-1α protein, which in turn upregulates glucose uptake and lactate production and promotes cancer growth [87].

### 3.1. GPC3 mRNA and Protein Expression

GPC3 mRNA expression has been investigated and correlated with HCC in several studies [40,41,42]. Hsu and colleagues studied 191 tumor samples from 154 patients with primary and/or recurrent HCC [40]. In total, 143 of 191 tumors (74.8%) were positive for GPC3 mRNA, whereas normal adult liver tissues were negative, suggesting that GPC3 mRNA can be used as an early tumor marker for HCC [40]. GPC3 mRNA levels were also evaluated in normal liver (15), focal nodular hyperplasia (7), liver cirrhosis (28), and HCC (30) samples. Weak expression was detected in some normal or cirrhotic samples and in all samples with focal nodular hyperplasia, whereas 75% of HCC samples had increased GPC3 mRNA levels [41]. Nakatsura and coworkers demonstrated 5-fold greater GPC3 mRNA expression in HCC cancer tissues than in noncancerous adjacent tissues in 16/20 samples [42].

The protein expression of GPC3 was also investigated. Capurro and colleagues examined 29 HCC and 58 non-HCC samples [43]. In total, 21/29 and 1/58 of the HCC and non-HCC samples, respectively, were GPC3 positive. Normal liver tissues were negative [43]. A similar correlation was found in other studies evaluating 31, 55, 58, 56, and 49 HCC samples, with GPC3 positivity of 68%, 50.9%, 94.8%, 84%, and 75.5%, respectively, while normal tissues were negative for GPC3 [39,45,46,47,49]. GPC3 expression correlated with poor prognosis after surgical resection, earlier recurrence of HCC, and increased risk of death for HCC patients, and was defined as an independent prognostic factor for poor disease-free survival in patients with early HCC [45,48]. A signal corresponding to the GPC3 protein was found in both the cytoplasm and membrane, with a granular pattern near the cell membrane [39,43,46,47,48,49].

In conclusion, the detection of GPC3 in immunohistochemistry allows discrimination between HCC tissue and normal adult liver tissue and between HCC and benign lesions thanks to its good sensitivity and specificity for HCC [39,43,45,46,47,48,49]. Its positivity does not correlate with tumor size; in fact, even small tumors were GPC3 positive, making it a good early diagnostic marker for HCC [39,43,45,46,49].

GPC3 has two important properties that make it an optimal target for drug delivery: it is specific to HCC cancer cells, and it is bound to the cell membrane and protrudes from the cellular compartment. Therefore, NPs equipped with an anti-GPC3 antibody can readily interact with the protein and be specific for HCC tumor cells.

### 3.2. Antibodies Targeting GPC3

Antibodies and antibody fragments are extensively used in clinical practice and as coating agents for targeting NPs [138,139,140].

#### 3.2.1. Condrituzumab (GC33)

GC33 IgG2a is able to recognize a region at the C-terminal subunit of GPC3. It showed antibody-dependent cell-mediated cytotoxicity (ADCC) action and tumor growth inhibition in a subcutaneous HepG2 and HUH7 mouse model and in an orthotopic model [141]. The humanized GC33 (Condrituzumab) showed the same efficacy in the HepG2 xenograft as GC33 [141]. In the first-in-man phase I study (NCT00746317), 20 patients with advanced HCC received condrituzumab, which was well tolerated and showed a preliminary treatment effect [142]. In the randomized phase II study (NCT01507168) on 185 previously treated HCC patients, the 125 patients treated with condrituzumab did not show clinical benefits [143]. In the phase 1b study (NCT00976170) on 41 patients, the association of condrituzumab and sorafenib was tolerated, but this combination treatment did not add other benefits over those already seen with sorafenib treatment [144].

#### 3.2.2. YP7

YP7 is a humanized IgG1 monoclonal antibody recognizing a region at the C-terminal subunit of GPC3 [145,146]. Preclinical studies on HepG2 tumor-bearing mice showed tumor growth inhibition. In addition, the humanized form of the antibody was able to induce ADCC and complement-dependent cytotoxicity (CDC) in GPC3-expressing tumor cells [145,146]. Humanized YP7 also demonstrated tumor growth inhibition in HCC xenograft nude mice [146].

#### 3.2.3. G12

1G12 antibody epitope is localized at the C-terminal subunit of GPC3 protein. 1G12 was able to recognize GPC3 in paraffin-embedded HCC tissue sections but not in non-malignant liver disorders [43]. The humanized form of 1G12 (H3K3) has been used for PET imaging once linked to ^89^Zn radioisotope. It was able to specifically bind GPC3-expressing patient-derived xenograft tumor and accumulate at the tumor mass, with minor uptake in the heart, brain, lungs, muscles, and bones [147].

#### 3.2.4. Single-Domain Antibodies

HN3 is a human heavy-chain variable domain antibody showing high affinity for GPC3 protein and able to recognize an epitope that spreads both at the N-terminal and C-terminal subunits [148]. It showed HepG2 and HUH7 tumor growth inhibition in xenograft mouse models without toxicities [148]. G2 is a single-domain antibody able to recognize GPC3 that has been labeled with gallium-68 and fluorine-18 to generate radiotracers in order to specifically detect subcutaneous HCC. Both gallium-68- and fluorine-18-labeled G2 specifically delineated HCC tumor in PET imaging. The addition of the albumin-binding domain significantly increased the uptake at the tumor site and decreased the accumulation in the kidneys [149].

#### 3.2.5. Bispecific Antibodies

ERY974 is whole humanized IgG bispecific antibody made by combining the anti-GPC3 GC33 antibody and the anti-CD3 CE115 antibody. Its sequence was modified to eliminate the interaction with FcγR-bearing cells and facilitate heterodimerization [150]. Mouse models confirmed its activity both for immunogenic and nonimmunogenic tumors, leading to tumor remission [150]. ERY974 has been evaluated for PET imaging using [^89^Zr]Zr-N-suc-Df-ER974, showing a specific tumor uptake that was grater in mice engrafted with human immune cells, suggesting a potential use of this labeled bispecific antibody for biodistributions studies in patients with the aim of drug development [151]. A phase I study (NCT05022927) is recruiting patients with HCC to evaluate the safety, tolerability, pharmacokinetics, anti-tumor effect, and biomarkers of ERY974 in combination with atezolizumab and bevacizumab [152].

#### 3.2.6. Anti-GPC3 Immunotoxins

Immunotoxins are composed of an antibody for the targeting and a toxin, usually from a plant or bacteria (*Pseudomonas aeruginosa* exotoxin A or diphteria toxin) [153,154]. scFv from YP7 has been bound to a truncated diptheria toxin, showing in vitro cytotoxic effects on HepG2 cells, with changes in morphology, G2 phase arrest, increased reactive oxygen species, and apoptosis [155]. HN3 has been used as immunotoxin bound to a truncated form of *Pseudomonas aeruginosa* exotoxin A in a single or bivalent form. It caused Hep3B-derived tumor regression in an HCC xenograft mouse model, with extended mice survival and no significant side effects [156]. The evaluation of HN3 as a target for the delivery of immunotoxins has also been studied using *Pseudomonas aeruginosa* exotoxin A with deimmunized domains. Three immunotoxins have been evaluated in vivo: HN3-mPE24 (furin cleavage linker and B cell deimmunized domain III), HN3-T19 (10 point mutations to reduce B and T cell antigenicity), and HN3-T20 (6 point mutations in domain III to reduce T cell antigenicity), all showing almost full regression of Hep3B-derived tumor in xenograft mice but also a decrease in mice weight. HN3-T20 showed the best results. HN3-T20 has been further modified by the addition of an albumin-binding domain, which resulted in tumor regression similar to mice treated with HN3-T20 but administration of 1/10 of the dosage, suggesting a possible clinical development [157].

#### 3.2.7. CAR-T Targeting GPC3

CAR-T cells are engineered T cells able to express a chimeric antigen receptor [158]. GC33 antibody has been used as a basis to create scFv in order to produce CAR-T cells able to recognize GPC3 protein [158,159,160]. An antitumor effect was observed in patient-derived xenograft mice treated with GPC3-CAR-T cells [158]. The co-expression of IL15 and IL21 improved both the expansion and persistence of GPC3-CAR-T cells in vivo, with an increase in the antitumor efficacy and mice survival [159]. scFv from GC33 and scFv for EGFR has been fused to produce a dual-targeting CAR-T and evaluated in orthotopic NOD/SCID mice. CAR-T presenting dual-targeting showed a higher increase in mice survival compared to mice treated with single-targeting CAR-T. Furthermore, blood analysis revealed a higher number of CAR-T dual-targeting cells with respect to single-targeting CAR-T, suggesting that dual-targeting CAR-T could survive better than the single-targeting CAR-T [160]. Several clinical trials are ongoing to test GPC3-CAR-T cells to treat HCC (NCT04121273, NCT05003895, NCT05155189, NCT05070156, NCT04951141, NCT05103631, NCT03198546, NCT02905188, NCT03884751, NCT03302403) [161]. Three trials have been completed (NCT03146234, NCT03980288, and NCT02395250) [161]. The phase I trial NCT03980288 used a fourth generation of CAR-T-cells to target GPC3 in combination with multi-tyrosine kinase inhibitors in patients presenting advanced HCC. Co-administration had a manageable and safety profile and demonstrated potential antitumor activity; however, cytokine release syndrome should be treated in all six patients [162]. The other phase I study of GPC3-CAR-T cells, NCT02395250, studied this therapy in Chinese patients with refractory or relapsed GPC3-positive HCC. Treatment was safe and had a potential antitumor effect when lymphodepletion was applied [163].

### 3.3. Anti-GPC3 Peptides

Another option for GPC3 targeting is the use of peptides, which show a decrease in immunogenicity, simplicity in performance, and low production costs [164]. One of the peptides produced is the L5 (RLNVGGRYFLTTRQ). It was chosen among seven candidates showing selective binding to GPC3-positive cells as opposed to others [164]. GBP (THVSPNQGGLPS) is another GPC3-binding option that showed in vivo accumulation at the HepG2 tumor site and discrimination between tumor and normal liver tissue in immunohistochemical analyses on samples from patients and healthy individuals [165]. TJ12P1 (DHLASLWWGTEL) was identified through a phage display screening showing GPC3-binding affinity [166]. In an in vivo study on HepG2 tumor-bearing mice, tumor accumulation of the labeled peptide was observed, which was higher compared to the accumulation in GPC3-low-expressing tumors. In addition, TJ12P1 was able to detect GPC3 protein in HCC patient-derived tumor tissue but not in healthy adult-derived liver tissue [166]. However, the specificities of L5 and TJ12P1 as targeting peptides for GPC3 protein have been questioned in an in vitro study [167].

### 3.4. Peptide Vaccines

Immunotherapeutic approaches also focused on the generation of tumor-specific CD8+ T cells able to recognize peptides of 8–11 residues deriving from intracellular proteins and presented associated with MHC class I molecules [168]. Sawada and coworkers generated two peptides based on GPC3 protein to study their effect as vaccines: EYILSLEEL and FVGEFFTDV [168,169,170]. These peptides can induce GPC3-reactive cytotoxic T lymphocytes without the activation of autoimmunity and the immunological effects in mice were dose dependent [168]. Subsequently, the two peptides were used in a phase I trial, showing a tolerable profile, a partial response in one patient out of 33, and a stable disease in 19 patients. A GPC3-specific cytotoxic T lymphocyte response was detected in 30 patients and its frequency was correlated with better overall survival [169]. The evaluation of the peptides moved towards a phase II study to evaluate them as adjuvant therapy for HCC [170]. A significantly lower recurrence rate was detected in GPC3-positive patients treated both with surgery and vaccination compared to GPC3-positive patients that only underwent surgery [170]. A phase I study using two peptide vaccine cocktails derived from GPC3, WDRPUH and NEIL3, was carried out [171]. The two cocktails showed good tolerability, with 14 patients showing stable disease and some tumor regression [171].

### 3.5. Radiopharmaceutical Therapy

GC33 has been exploited for radiotherapy coupled with the radionuclide actinium-225 to synthesize the radioconjugate [^225^Ac]Ac-Macropa-GC33 [172]. HepG2 tumor-bearing mice were treated with two dosages of radioconjugate and tumor delay was observed. However, significant toxicities have also been detected in all treated mice, suggesting the need for optimization in the activity amount and administration [172]. An antibody targeting GPC3 has been used as radioconjugate with ^227^Th(NO_3_)_4_ to synthetize ^227^Th-octapa-αGPC3 [173]. An orthotopic mouse model presenting HepG2 tumor was set up and mice were treated with the radioconjugate ^227^Th-octapa-αGPC3, showing accumulation at the tumor site and a tumor killing effect [173].

## 4. Nanoparticles Composed of Organic-Based Nanomaterials

Organic NPs have been widely studied for cancer treatment, showing antitumor effects, improved bioavailability, biodegradability, controlled release, and tunable structures [32,35,174]. Size, surface properties, and drug encapsulation and release are fundamental properties for an optimized delivery approach [32,35,174]. Among the organic NPs, polymeric NPs and liposomes can be found as conventional NP platforms [88,175]. Polymeric NPs can be produced from natural polymers (such as cellulose, peptides, proteins, chitosan, etc.), synthetic polymers (such as PLA, PLGA, etc.), or microbial fermentation polymers (such as polyhydroxybutyrate) [88]. Abraxane was the first polymeric NP approved by FDA [88]. Liposomes are small artificial vesicles with a spherical shape made of cholesterol and natural non-toxic phospholipids whose bilayer components determine their degree of rigidity. They are included in many commercial formulations and are present in most nanotechnology-based drugs that are widely used in oncological hospitals (e.g., doxil) [176].

### 4.1. The Most Common Matrices

In Table 2, the principal characteristics of NPs that can be employed for cancer treatment, including the advantages and disadvantages of the composed nanomaterials are summarized.

### 4.2. Size and Surface Characteristics

Size and size distribution are the most important properties of NPs because they affect NPs’ stability, distribution, toxicity, targetability, drug loading, and release [182]. It is believed that the lower size limit for NPs in cancer treatment should be 10 nm, the threshold for first-pass elimination by the kidneys [32]. The upper limit is not so well defined. It is known that tumor vessels are irregular, have discontinuous epithelium without basement membranes, and are characterized by fenestrations ranging from 200 to 2000 nm [32,183]. These features, together with the additional impaired lymphatic system, result in a leaky vasculature through which macromolecules can leak from the vessels and accumulate in the tumor tissue. This phenomenon is referred to as enhanced permeability and retention (EPR) effect [32,183]. Smaller NPs can easily extravasate through fenestrated vessels but can also pass-through normal blood vessels to reach healthy tissue. Larger NPs, on the other hand, are more difficult to extravasate but preferentially accumulate in tumor tissues due to the EPR effect [35,184].

Once NPs are in a biological system, the properties of their surfaces and the nature of their core determine the type of proteins with which they interact. These proteins are referred to as protein corona and can influence the fate of NPs, such as their recognition by phagocytic cells and their residence time in blood [184,185,186]. Hydrophobic, high-positive, or high-negative NPs are recognized by the reticulo-endothelial system (RES) as non-self, resulting in their elimination from the bloodstream by the liver and spleen [32,35,182]. To avoid this, NPs must prevent or at least reduce their opsonization. This can be achieved by coating the NPs with polyethylene glycol (PEG), which makes the NPs more hydrophilic and increases their solubility, stability, and circulation time [35,182]. The length and surface density of PEG determine the adsorption of plasma proteins and the uptake rate by polymorphonuclear cells, which represents a new level in the formulation of NPs [186].

### 4.3. Drug Encapsulation and Release

Drugs can be divided into hydrophobic and hydrophilic. Solubilization of hydrophobic drugs in a biological aqueous medium can be difficult. They also tend to aggregate after intravenous administration, which can lead to emboli and local toxicity [34]. On the other hand, hydrophilic drugs are poorly taken up by cells due to the obstacle of lipid-rich hydrophobic cell membranes; they are also subjected to hydrolytic and proteolytic degradation and therefore have a short half-life in blood [34].

Encapsulation of drugs in NPs prevents drug degradation, facilitates the delivery of both hydrophobic and hydrophilic drugs, increases their bioavailability, and allows controlled release in a disease-specific microenvironment, thereby reducing toxicity in normal tissues [32,34,35,182]. Two methods are possible for drug encapsulation: incorporation or adsorption. In the first case, the drug is already present during the formation of the NPs while in the second case, the preformed NPs are incubated in the presence of a high concentration of drug, which is then absorbed by the NPs [182]. A high drug loading capacity is required for a successful delivery system, as this allows the matrix content to be reduced for each administration [182,187].

Drug release is an important characteristic for an effective delivery approach. The release correlates with the solubility of the drug, the desorption of the drug adsorbed inside the NPs or bound to the surface of the NPs, the diffusion of the drug through the NPs matrix, the erosion or degradation of the NPs matrix, and finally the combination of erosion and diffusion processes [182,187]. If the diffusion process is faster than the matrix degradation, the release depends on diffusion; otherwise, on degradation [187]. Burst release must be avoided to prevent off-target toxicity. Long-term controlled release is preferred and is one of the goals for therapeutic treatment by NPs [185].

Drug release can also be triggered by internal (stress, pH changes, redox, enzymes, hypoxia) or external stimuli (ultrasound, temperature, light, magnetic forces), resulting in a fine-tuned release system [35,184,188]. Examples of stimuli-responsive NPs for drug release for the treatment of HCC are summarized in Table 3.

### 4.4. Targeting and Anti-GPC3 NPs Drug Delivery

**Figure 4:**
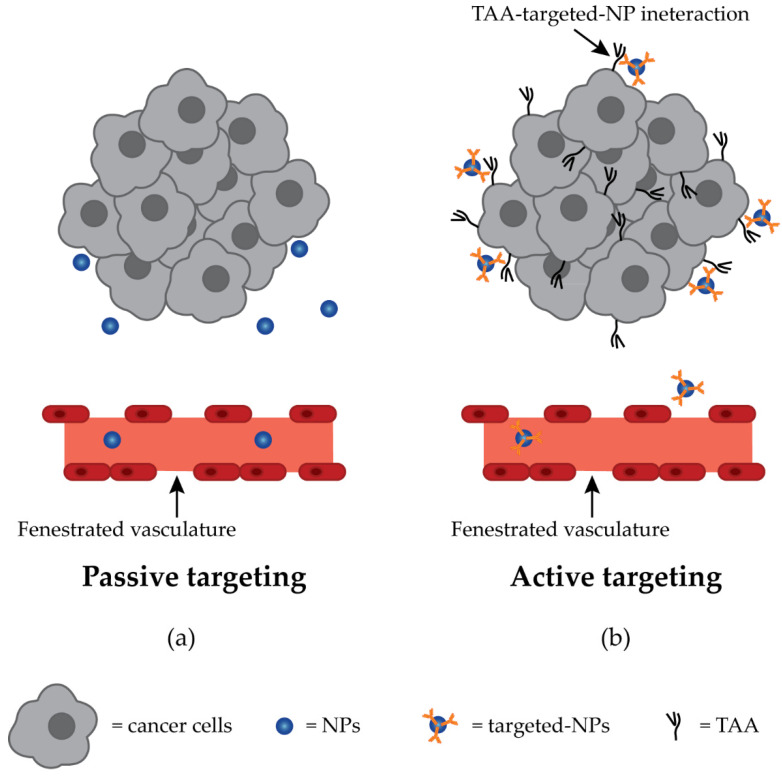
Types of targeting in a drug delivery approach using NPs. (**a**) Leaky and fenestrated vasculature allows the NPs to leave blood circulation and reach the tumor site by passive targeting. (**b**) Active targeting relies on the presence of ligands on the NPs surface, which interact with their TAA, increasing NPs’ specificity.

Once administered, NPs can reach their target in two ways: passive targeting or active targeting (Figure 4) [34,182]. The first route uses the aforementioned EPR effect, pH change, or infusion through a catheter to reach the tumor. The second method is achieved by binding a tissue- or cell-specific ligand to the surface of NPs [34,182].

NPs’ delivery based solely on the EPR effect may lead to off-target distribution to organs that normally have a fenestrated vasculature, such as the liver and spleen [35]. Therefore, active targeting is required for a focused approach, which allows the interaction of NPs with cell surface receptor proteins or TAAs and is exploited by different types of ligands: peptides, nucleic acids, small molecules, carbohydrates, proteins, enzymes, antibodies, and antibody fragments [32,34,35,184]. The presence of the ligand on the surface of NPs increases the binding affinity and internalization in the cell through receptor-mediated endocytosis. However, in solid tumors, NPs still need to utilize the EPR effect to reach the tumor tissue [34].

#### 4.4.1. Polymeric NPs

TPGS-*b*-PCL NPs loaded with sorafenib have been exploited to study their effects in in vitro and in vivo HCC models. In both cases, researchers used the anti-GPC3 9C2 antibody to redirect NPs towards GPC3-positive HCC cells [57,196]. In the study of Gan and colleagues, the presence of the antibody increased NPs’ uptake in HepG2 cells compared to non-targeted NPs. In addition, the MTT assay showed a higher cytotoxicity of GPC3-targeted NPs loaded with sorafenib compared to the non-targeted ones and free drug. Results were confirmed in the in vivo HCC mouse model, where mice presenting HepG2 tumor were treated with the targeted NPs and showed a greater sorafenib-derived antitumor effect, with suppression of tumor growth, compared to non-targeted NPs and free sorafenib [57]. Additionally, Tang and colleagues demonstrated the ability of TPGS-*b*-PCL NPs loaded with sorafenib and coated with anti-GPC3 antibody to increase cellular uptake and cytotoxicity compared to non-targeted NPs [196]. They also detected a downregulation of the anti-apoptotic molecule MCL-1 and promotion of cytochrome C release with consequent cell apoptosis. An in vivo HepG2 xenograft mouse model revealed significant inhibition of tumor growth in the presence of targeted NPs loaded with sorafenib compared to non-targeted NPs and free sorafenib [196]. GC33 has been used as targeting agent of PEG-*b*-PLGA NPs loaded with sorafenib [197]. In vitro studies revealed that GPC3-targeted NPs without loading were able to inhibit HepG2 proliferation, increasing the proportion of cells at G1 phase. In addition, downregulation of cyclin D1 was observed. These NPs were also able to inhibit the transduction of the proliferation signal dependent on Wnt3a to inhibit epithelial mesenchymal transition, thus attenuating cell migration. In the in vivo study on HepG2 and HUH7 tumor-bearing mice, an inhibition of tumor growth once targeted-NPs loaded with sorafenib were administered, an increase in mice survival, and no toxic effects compared to free drug and untargeted NPs were observed [197].

#### 4.4.2. Liposomes

Liposomes coated with the GPC3-targeting peptide G12 (FITC-TJ12P1) and loaded with sorafenib and IR780 iodide were able to detect early H22 tumors in murine models with more significant sensitivity compared to liposomes coated with folic acid [198]. In addition, liposomes targeting GPC3 showed antitumor efficacy, which was grater when the external stimulus coming from laser light was added and with respect to folic-acid-targeted liposomes [198]. Magnetic liposomes coated with anti-GPC3 and anti-EpCAM antibodies have been used as drug carriers for Lenvatinib [199]. In vitro and in vivo results showed the specificity of the magnetic liposomes towards HCC cells and the effective aggregation of drugs on tumor cell surfaces. In vitro study also revealed the potential use of the magnetic liposomes for magnetic resonance (MR) imaging [199]. Liposomes coated with anti-GPC3 and anti-VCAM1 antibodies have been evaluated to deliver sorafenib and digitoxin (GV-Lipo/SF/DT) to treat HCC [200]. GV-Lipo/SF/DT was able to target circulating tumor cells, dissociate their clusters, prevent the formation of circulating tumor cell-neutrophil clusters, and inhibit tumor spread. The liposomes showed an antitumor effect in H22 tumor-bearing mice, which was greater in the group treated with GV-Lipo/SF/DT compared to mice treated with free digitoxin, or with liposomes presenting only one targeting antibody or no targeting at all [200]. In addition, the combination of both sorafenib and digitoxin in the liposomes showed a greater antitumor effect compared to single drug liposomes thanks to a synergistic effect that acts by blocking cells at G1 phase and loosening cell–cell junctions, facilitating deeper tumor penetration by liposomes [200]. The antitumor effect was also observed in an orthotopic mouse model, with GV-Lipo/SF/DT showing the greatest effects. GV-Lipo/SF/DT was also able to greatly prevent pulmonary metastases in mice intravenously injected with H22 cells [200].

#### 4.4.3. Imaging

Conventional contrast-enhanced agents for MR imaging do not reach high specificity for HCC while molecular MR imaging through magnetic NPs showed promising results [201]. The use of peptides as targeting agents for MR could overcome some limitations of antibodies, such as immunogenicity and high costs [201]. The L5 peptide bound to biotin has been used as a pretargeting agent to block ultrasmall superparamagnetic iron oxide (USPIO) NPs bound to streptavidin in GPC3-positive HepG2 cells. Pretreatment with L5-biotin peptide determined a significant decrease in signal intensity once mice were treated with USPIO NPs bound to streptavidin and a marked deposition of iron was also detected [201]. Another GPC3-binding peptide, GBP, has been used for enhanced photo-sonotheranotics of HCC. Its coupling with NH_2_-PEG_36_-COOH and the croconaine dye (CR-1) allowed the synthesis of a theranostic agent (CR-PEG-GBP) that switches from a monomer state in a physiological environment to an NP form under the acidic pH present at the tumor site [202]. The ability to switch from one state to the other allowed an accumulation at the tumor site, being in the NP form, and easy excretion from normal tissues, being in the monomeric form. Imaging using NIR-II light demonstrated that CR-PEG-GBP was able to highlight the HepG2 tumor in a mouse model with a high tumor-to-tissue ratio and an increased tumor retention compare to CR-1 alone. CR-PEG-GBP was also validated for photoacoustic signaling. The combination of photothermal therapy and sonodynamic therapy showed the best results in tumor growth inhibition and increased mice survival [202]. The *N*-maleoyl-*β*-AYFLTTRQ GPC3-binding peptide has been used to functionalize silica NPs for ultrasound molecular imaging [203]. Their biocompatibility was confirmed by an MTT test on HepG2 cells and cellular uptake was greater for targeted-NPs compared to non-targeted ones and it was correlated with GPC3 expression [203]. It should be noted that the specificity of L5 and TJ12P1 for GPC3 protein has been questioned [167].

## 5. Conclusions

Two major interrelated problems of HCC are diagnosis and treatment. For patients who do not participate in surveillance programs, the diagnosis is usually made at a late stage when malignancy becomes symptomatic and curative treatments are no longer available. For these patients, systemic therapies are the only available options, with the attendant side effects that these treatments bring. Several TAAs are overexpressed in HCC to meet the increased nutrient demand of tumor cells caused by their enhanced proliferation, such as receptors for asialoglycoproteins, transferrin, and folic acid, or correlate with the exacerbation of tumor features, such as MUC1, CEA, and TEM1. An interesting membrane protein present in HCC cells is the oncofetal protein GPC3, which interacts with proliferative signaling pathways such as WNT. It is specifically re-expressed in HCC tissues, also in the early stages, but barely expressed in healthy adult tissues and in cirrhotic or premalignant liver lesions, making it a specific TAA for the malignancy. It is anchored to the cell membrane and protrudes outside the cellular compartment, making it a promising target for a therapeutic approach. A lot of efforts have been made to exploit GPC3 for targeting strategies in order to improve diagnostic imaging and treatment of HCC. One of these methods takes advantage of NPs. Drug delivery platforms that employ NPs have been demonstrated to be capable of overcoming the pharmacokinetic limitations that are generally associated with the conventional drug formulations used for the treatment of cancer, including HCC. The proposed NPs have been demonstrated to enhance the solubilization of drugs and their circulation lifetimes. In addition, the possibility of conjugating the NP surface with a targeting agent specific for a TAA allows the development of a drug delivery system with a further increased capability to reach the tumor. In particular, NPs coated with anti-GPC3 targeting agents showed great specificity for HCC tumor cells, with the consequent improvement in imaging and drug delivery both in vitro and in vivo compared to the non-targeted counterparts and free drugs. Moreover, the possibility of delivering high drug amounts specifically to tumor cells could overcome drug resistance, usually caused by low drug concentration treatments, and accelerate tumor cell death. Another possibility of treating HCC is based on its different vascularization compared to liver. HCC presents almost exclusively arteries while liver is characterized by a dual supply of both portal and arterial components. Injection of NPs, as in TACE treatment, could take advantage of this situation. An imaging assessment in a pre-treatment step could allow the identification of the best arteries in order to focus the NPs injection, and the consequent treatment, only at HCC tissue sparing healthy liver. In the end, the advantages that NPs have in terms of their formulations and modifications must deal with the elimination through the RES system and lung vasculature accumulation. In the first case, NPs are subjected to protein adsorption on their surfaces once they reach the blood circulation. Protein adsorption promotes opsonization, aggregation, and rapid clearance from the bloodstream through the RES system in the liver and spleen. In the second case, the accumulation of NPs at pulmonary vasculature may lead to pulmonary inflammation and it depends on the route of administration, number of administrations, and NPs composition. For these reasons, even if NPs have been used for years in a preclinical context, few of them have been translated into the clinic. To conclude, although NPs have great potential, many efforts still need to be invested to move from preclinical practice to clinical treatments.

## Figures and Tables

**Table 1:** Systemic therapies for advanced HCC.

Drug	Molecule Type	Line Treatment and Year of Approval
Sorafenib	Multi-tyrosine kinase inhibitor	First line, standard of care until 2020 Approved in 2008 [66,67]
Regorafenib	Multi-tyrosine kinase inhibitor	Second line Approved in 2017 [66,68]
Lenvatinib	Multikinase inhibitor	First line Approved in 2018 [66,69,70]
Atezolizumab + bevacizumab	Anti-PD-L1 antibody + anti-VEGF antibody	First line, standard of care Approved in 2020 [66,71,72,73,74]
Nivolumab	Anti-PD1 antibody	Second line Approved in 2017 [66,75,76]
Pembrolizumab	Anti-PD1 antibody	Second line Approved in 2018 [66,77]
Cabozantinib	Tyrosine kinase inhibitor	Second line Approved in 2019 [66,78,79]
Ramucirumab	Anti-VEGFR-2 antibody	Second line Approved in 2019 [66,80]
Nivolumab + ipilimumab	Anti-PD1 antibody + anti-CTLA-4 antibody	Second line Approved in 2020 [66,81]
Durvalumab + Tremelimumab	Anti-PD-L1 + Anti-CTLA-4	FDA grants priority review to AstraZeneca’s Biologics License Application April 2022 First line [82,83,84,85]
Durvalumab	Anti-PD-L1	Supplemental Biologics License Application has been submitted to FDA April 2022 First line [82,83,84,85]

**Table 2:** The most common matrices for NPs synthesis and their properties.

Matrix Component	Features	Advantages	Disadvantages
poly(lactic acid) (PLA), poly(lactic-co-glycolic acid) (PLGA)	Synthetic origin. Biocompatible. Biodegradable. Non-toxic. Negatively charged. [177]	Synthesis can be done with different molecular weights and lactic:glycolic acid ratios. Different NPs shapes can be made. Sustained release of loaded drugs. Surface modifications are possible. PLA-based NPs have been proposed to increase the oral bioavailability of poorly water-soluble drugs. [177]	Poor drug loading. Some formulations have an initial high burst drug release. [177]
Albumin (AL)	Natural origin. Biocompatible. Biodegradable. Non-immunogenic. Water-soluble. Negatively charged. [178,179]	Rich in functional groups for ligand/drug binding. AL-based NPs easily cleared in vivo. It is naturally internalized in tumor stroma through the gp60 pathway. High levels of albumin can be supplemented into the body without or with low effects. [178,179]	Some formulations need toxic cross-linking with drugs to increase NPs stability and avoid a burst release. [179]
Chitosan (CS)	Natural origin. Biocompatible. Biodegradable. Non-toxic. Positively charged. [180]	It presents hydroxyl and amine functional groups for the addition of crosslinking agents. Positive charge allows CS to attach to cells, tending to accumulate in negatively charged cancer cells, and increase cellular uptake. pH sensibility prevents drug release at physiologic pH (~7.4) and increases it in the acid tumor environment. [180,181]	Low solubility at physiologic pH. Fast dissolution in stomach. [180]
Cholesterol and lipid layer (Liposomes)	Natural origin. Non-toxic. Biocompatible. Biodegradable. Non-immunogenic. Sphere-shaped. [176]	Modification of the lipid layer structure to imitate biophysical characteristics of cells. Reduce toxic drug exposures of sensitive tissues. [176]	Low solubility. Short half-life. Possible leakage and fusion of drug/molecules encapsulated. [176]

**Table 3:** NPs with stimuli-responsive drug release.

Stimulus	NPs Type	Properties	In HCC
pH	Anionic: Poly(aspartic acid) Poly(acrylic acid) Poly (methacrylic acid) Poly-sulfonamides Cationic: Poly(b-amino ester) Poly(N,N-dimethylamino ethyl methacrylate) poly(L-histidine) [189]	Ionizable groups in NPs structure. Imbalance in hydrophilic-hydrophobic equilibrium at low pH. Disruption of NPs structure with the consequent drug release. [189]	poly(b-amino ester) copolymer NPs loaded with doxorubicin and curcumin had an enhanced and rapid release in HCC acidic environment (pH 5.8) and an increase in cellular uptake. In vivo NPs showed an increased tumor weight inhibition compared to free drugs [190]
Redox	Disulfide-linked [188]	Exploit the high levels of glutathione in cancer tissues. Presence of disulfide bonds, which are disrupted in presence of glutathione. [188]	NPs made of a polymer with isocyanate and bis(2-hydroxyethyl)-disulfide with the terminal end pegylated. Triptolide were loaded into NPs. Triptolide is rapidly release in reductive conditions with improved antitumor efficacy and low toxicity. [191]
Ultrasound (US)	Nanobubbles (NBs) [192,193]	External biodegradable shell. Inner core of vaporizable compound or gas (perfluorocarbons or sulfur hexafluoride). US delivery based on bubble cavitation and increased cell permeability. [192,193]	Lina Du and colleagues investigated the effect of doxorubicin-loaded PLGA-mPEG NBs in the treatment of mice with subcutaneous H22 tumor (mouse hepatocellular carcinoma cell line). Doxorubicin-NBs showed a reduction in tumor growth compared to mice treated with saline. This effect was further enhanced by the additional external stimulus of US. Lipidic-shelled NBs loaded with 5-fluorouracil showed antitumor effect in HepG2 tumor-bearing mice, with a greater extent once US were administered. [194,195]

## Data Availability

Not applicable.

## Abbreviations

ADCC = antibody-dependent cell-mediated cytotoxicity; AFP = alpha-fetoprotein; AL = albumin; ASGPR = asialoglycoprotein receptor; BCLC = Barcelona Clinic Liver Cancer; CDC = complement-dependent cytotoxicity; CEA = carcinoembryonic antigen; CR-1 = croconaine-1 dye; CS = chitosan; ECOG = Eastern Cooperative Oncology Group; EpCAM = epithelial cell adhesion molecule; EPR = enhanced permeability and retention effect; FDA = Food and Drug Administration; FR = folic acid receptor; GPC1, GPC2, GPC3, GPC4, GPC5 and GPC6 = glypican 1, 2, 3, 4, 5, 6; GPI = glycosylphosphatidylinositol; HBV = hepatitis B virus; HCC = hepatocellular carcinoma; HCV = hepatitis C virus; HIF-1α and HIF-2α = hypoxia inducible factor-1α and -2α; HS = heparan sulfate; MAGE = melanoma antigen gene; MDK = midkine; MR = magnetic resonance; MUC1 = mucin 1; NBs = nanobubbles; NPs = nanoparticles; NY-ESO-1 = new york esophageal squamous cell carcinoma-1; PEG = polyethylene glycol; PLA and PLGA = poly(lactic acid) and poly(lactic-co-glycolic acid); PRL-1 and PRL-3 = phosphatases of regenerating liver-1 and -3; PSMA = prostate specific membrane antigen; RES = reticuloendothelial system; SR-B1 = scavenger receptor class B type 1; SSX-2 = synovial sarcoma X breakpoint 2; TAA = tumor-associated antigen; TACE = transarterial chemoembolization; TEM1 = tumor endothelial marker 1; TERT = telomere reverse transcriptase; TfR1 = transferrin receptor 1; US = ultrasound; USPIO = ultrasmall superparamagnetic iron oxide.
